# Morphogenetic mechanism of the acquisition of the dinosaur-type acetabulum

**DOI:** 10.1098/rsos.180604

**Published:** 2018-10-17

**Authors:** Shiro Egawa, Daisuke Saito, Gembu Abe, Koji Tamura

**Affiliations:** 1 Department of Ecological Developmental Adaptability Life Sciences, Graduate School of Life Sciences, Tohoku University, 6-3, Aza-aoba, Aramaki, Aoba-ku, Sendai 980-8578, Japan; 2 Frontier Research Institute for Interdisciplinary Sciences (FRIS), Tohoku University, 6-3, Aza-aoba, Aramaki, Aoba-ku, Sendai 980-8578, Japan

**Keywords:** dinosaur, acetabulum, joint development

## Abstract

Understanding morphological evolution in dinosaurs from a mechanistic viewpoint requires the elucidation of the morphogenesis that gave rise to derived dinosaurian traits, such as the perforated acetabulum. In the current study, we used embryos of extant animals with ancestral- and dinosaur-type acetabula, namely, geckos and turtles (with unperforated acetabulum), and birds (with perforated acetabulum). We performed comparative and experimental analyses, focusing on inter-tissue interaction during embryogenesis, and found that the avian perforated acetabulum develops via a secondary loss of cartilaginous tissue in the acetabular region. This cartilage loss might be mediated by inter-tissue interaction with the hip interzone, a mesenchymal tissue that exists in the embryonic joint structure. Furthermore, the data indicate that avian pelvic anlagen is more susceptible to paracrine molecules, e.g. Wnt ligand, secreted by the hip interzone than ‘reptilian’ anlagen. We hypothesize that during the emergence of dinosaurs, the pelvic anlagen became susceptible to the Wnt ligand, which led to the loss of the cartilaginous tissue and to the perforation in the acetabular region. Thus, the current evolutionary-developmental biology study deepens our understanding of morphological evolution in dinosaurs and provides it with a novel perspective.

## 1. Introduction

**Figure 1.**
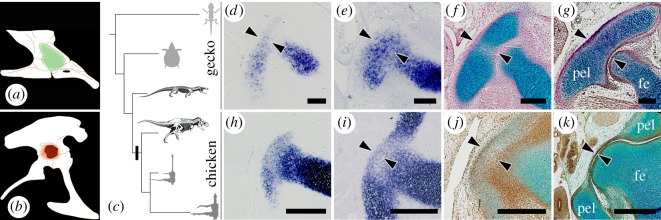
Evolutionary and developmental processes of the acetabulum. Pelvis with (*a*) unperforated or (*b*) perforated acetabulum (green and red region, respectively), drawn based on [7] and [8], respectively. (*c*) The phylogenetic relationships between animals used in the current study (grey silhouettes) and extinct archosauromorphs (skeletons are reprinted from [2,8] with permission). The bold black bar indicates the acquisition of the perforated acetabulum. (*d–g*) The pelvic anlagen of gecko (NR13, 15, 20 and 30, respectively). (*h–k*) The pelvic anlagen of chicken (HH27, 28, 30 and 36, respectively). The cartilaginous tissue was stained by *in situ* hybridization with aggrecan (*d,e,h,i*) or alcian blue (*f,g,j,k*). pel, pelvis; fe, femur; arrowheads, the acetabular region. Scale bars, (*d–f*), 100 µm; (*g–j*), 300 µm; (*k*), 500 µm.

The derived hind limb gait of dinosaurs is one of their key innovations, with their legs held in a parasagittal position, while their ancestors had a sprawling gait [1–4]. This was clearly advantageous for dinosaurs as it improved their locomotion [1,3,5] and allowed breathing while running [6]. The main rationale for this gait difference is the hip joint morphology, wherein the femur articulates with the pelvis at the acetabulum. While the ancestral state of the acetabulum is unperforated, the acetabulum became perforated with the emergence of dinosaurs (figure 1*a–c*). The perforated acetabulum of dinosaurs may have been covered by a membrane as in extant birds [9,10] to relieve pressure within the joint cavity associated with the parasagittal gait [11,12]. Considerable efforts, based on fossil record analysis and comparative anatomy, have been dedicated to the characterization of this morphological transition and its functional significance [1,3]. However, no previous study focused on the evolutionary changes involved in the morphogenesis of the perforated acetabulum—such knowledge would provide critical mechanistic insights into morphological evolution.

In the current study, to address the developmental mechanism underlying the acquisition of the dinosaur-type acetabulum, we undertook comparative and experimental analyses of acetabular morphogenesis, using embryos of extant ‘reptiles’ (i.e. non-avian sauropsids) and birds, which have ancestral (unperforated) and dinosaur-type (perforated) acetabula, respectively.

## 2. Material and methods

### 2.1. Animals

The experiments involved fertilized eggs of the following animals: gecko (*Paroedura picta*; eggs were collected from adults maintained in the laboratory, incubated at 28–29°C, staged according to [13] (NR stage, which is equivalent to dpo in [13])); turtle (*Pelodiscus sinensis*; Daiwa Youshoku, Japan, embedded in wet sand and incubated at 30°C, staged according to [14] (TK stage)); ostrich (*Struthio camelus*; Niseko Ostrich Farm and KO-COOP, Japan, incubated at 37°C, staged according to [15] (GA stage)); quail (*Coturnix japonica*; Motoki Corporation, Japan; incubated at 38.5°C, staged according to [16] (HH stage)) and chicken (*Gallus gallus domesticus*; wild-type, Iwaya Poultry Farm, Japan; homozygous and heterozygous limbless mutant, UC Davis Avian Science Facility, USA; incubated at 38.5°C, staged according to [16] (HH stage)).

### 2.2. *In situ* hybridization

We performed conventional *in situ* hybridization (ISH) on tissue sections as described in a previously published protocol [17], except that we omitted the proteinase K treatment and the subsequent fixation steps were omitted. We used partial or full-length clones as riboprobes, which we obtained by reverse transcription–PCR, using the primers listed in the electronic supplementary material, and sequenced. The gecko-chordin probe was kindly gifted by Dr H. Kiyonari (Riken, Japan) [18].

### 2.3. Histological analysis

We fixed embryos with 10% neutral buffered formalin or Bouin's solution, dehydrated them using an ethanol series, embedded them in a paraffin block after passing them through a xylene/paraffin series and sectioned them to 10–20 µm thickness. After the final wash with xylene and rehydration in an ethanol series, we stained the slices using the Maeda resorcin-fuchsin solution, Weigert's iron haematoxylin, 1% alcian blue in 70% ethanol and 3% acetic acid, and 0.1% eosin Y in 70% ethanol or van Gieson solution. This was followed by dehydration in an ethanol series, treatment in xylene and sealing with EUKITT. We photographed the samples using an upright microscope (Olympus, BX51).

### 2.4. Limb bud culture

We isolated hindlimb buds from HH22–26 chickens in Tyrode's solution/penicillin–streptomycin (PS) (Gibco). We placed the explants on the blood vessel of the chorioallantoic membrane (CAM) of e8–10 chicken eggs, added 0.3–0.5 ml Tyrode's solution/PS, sealed and incubated them at 38.5°C for 4.5 days. The explants were then fixed with Bouin's solution, stained with 0.1% alcian blue in 70% ethanol and 1% HCl, dehydrated in an ethanol series and cleared in methyl salicylate. Only samples in which the pelvic cartilage developed were counted.

### 2.5. Co-culture experiments: skeletal element + skeletal element

We used the following skeletal anlagen: the pelves of chicken (HH30–32), ostrich (GA13–14), turtle (TK11–19) and gecko (NR6–24); the femora of quail (HH30–32), ostrich (GA13), turtle (TK20) and gecko (NR17–22); the tibia of quail (HH30); the humerus of quail (HH31–33) and the pectoral girdle of quail (HH30). Agarose gel (2%) was mixed with black ink, set and cut into approximately 1 mm^3^. We isolated partial limb buds (for TK11–15 and NR6–15 pelves; stages before pelvic anlagen development or stages that are difficult to isolate) or skeletal elements (for later stages) in Tyrode's solution/PS and anchored to each other with tungsten needles. For limb buds, we anchored the long bone anlagen to their medial side. We placed the explants on a CAM blood vessel of e8–10 chicken eggs, added 0.3–0.5 ml of Tyrode's solution/PS, sealed and incubated them at 34°C for 8–10 days (turtle/gecko femora + chicken pelvis, and quail femora + turtle/gecko pelves) or 38.5°C for 4.5–5 days (others). We visualized the skeletons in the same way as the limb bud cultures. We only counted samples in which both the anlagen developed and the combined elements were still in contact—occasionally confirmed with histological analysis.

### 2.6. Expression vectors and transfection-stable Cos7 cell lines

We isolated the ORFs of chicken Noggin and Wnt9A by PCR using the following primers: Noggin: 5′-attacgcgtATGGATCATTCCCAGTGCCTTGTGACTATATACGC-3′ (forward) and 5′-aatgatatCTAGCAGGGCACTTGCACTCCGCGATGATGGGGTACTGGA-3′ (reverse); Wnt9A: 5′-attacgcgtATGCTGGATGGACACGTCCTGCTGGGATGGCTCTCCTCCT-3′ (forward) and 5′-attgataTCAGTCTTTACAGGTGTAAACCTCCTCTCTCTGGGTGCAC-3′ (reverse). We subcloned amplified fragments into the *Mlu*I-*Eco*RV sites of pT2A–BI–TRE–Gap43–TdTomato to obtain pT2A–Noggin–BI–TRE–Gap43-TdTomato and pT2A-Wnt9A–BI–TRE–Gap43–TdTomato. pT2A–BI–TRE–Gap43–TdTomato was gifted by Dr Y. Takahashi (Kyoto University).

We maintained Cos7 cells at 38°C in Dulbecco's modified Eagle medium (DMEM) (Gibco) supplemented with 10% fetal bovine serum (FBS) and PS. pT2A–BI–TRE–Gap43–TdTomato, pT2A–Noggin–BI–TRE–Gap43–TdTomato or pT2A–Wnt9A–BI–TRE–Gap43–TdTomato was co-transfected with pCAGGS–T2TP and pT2 K–M2–IRES–NeoR into Cos7 cells using Lipofectamine 2000 (Invitrogen) as previously described [19]. We cultured the cells in the G418-containing medium for more than two weeks.

### 2.7. Co-culture experiments: skeletal element + cell aggregate

After we harvested Cos7 cells with 0.05% trypsin and suspended in DMEM/10%FBS/PS, we adjusted their concentrations to 2.47–3.54 × 10^6^ cells ml^−1^ and cultured them in 2.5 µl as hanging drops overnight at 38°C under 5% CO_2_. Cell aggregates were anchored to the skeletal elements or limb buds isolated as described above (chicken (HH30–32), turtle (TK15-18) and gecko (NR17–22) pelves, and chicken (HH31–32) pectoral girdle). We placed the explants on a CAM blood vessel of e8–10 chicken eggs, added 0.5 ml of Tyrode's solution/PS/doxycycline mixture and sealed and incubated the host eggs at 38.5°C for 4.5–5 days. We examined the samples under a stereoscopic microscope (Leica, M165FC). For explants in which fluorescence was observed at the anchoring point, we visualized the skeletons in the same way as the limb bud cultures. We only counted samples in which the anlagen developed.

## 3. Results

### 3.1. Avian acetabular perforation results from the loss of cartilaginous tissue in the acetabular region of the pelvic anlagen during embryogenesis

To elucidate the morphogenetic process resulting in acetabular perforation, we first compared the development of pelves, which arise from the cartilaginous anlagen, in gecko and chicken embryos. During gecko embryogenesis, cartilaginous tissue was observed in the acetabular region throughout development, from the incipient stage of the pelvic anlagen (indicated by the ISH signal of aggrecan and alcian blue; figure 1*d–g*, arrowheads). During chicken embryogenesis, the incipient pelvic anlagen was observed in continuity with the femoral anlagen (figure 1*h*) as reported in perforated [20] and unperforated [21] acetabular developments. In the following stage, similarly to gecko's development, cartilaginous tissue was apparent in the acetabular region at the earliest stage when the separation of the pelvic and femoral anlagen was observed, although it was thin (figure 1*i*, arrowheads). In the chicken, this acetabular cartilaginous tissue was gradually lost and replaced by membranous connective tissue at the subsequent stages (figure 1*j,k*). These indicate that the acetabular perforation stems from the disappearance of the cartilaginous tissue in the acetabular region during embryogenesis.

### 3.2. Interaction between the pelvic anlagen and the hip joint interzone and/or femoral anlagen mediates avian acetabular perforation

To elucidate the mechanism underpinning the loss of acetabular cartilaginous tissue in the pelvic anlagen, we next focused on the inter-tissue interactions among the embryonic hip joint components.

**Figure 2.**
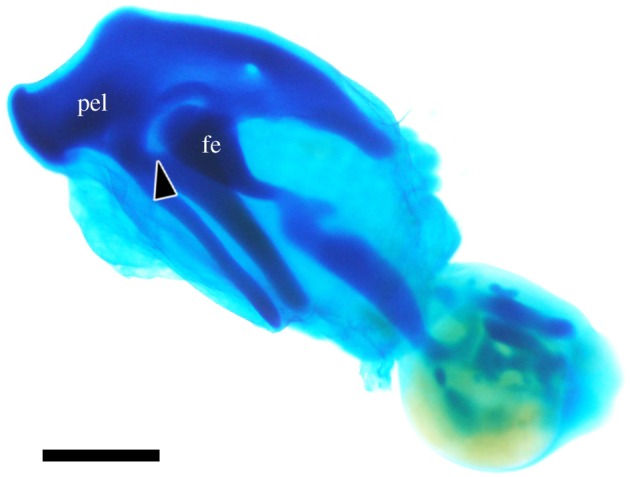
Hindlimb bud culture. pel: pelvis; fe: femur; arrowhead: acetabular foramen. Scale bar, 1 mm.

First, we analysed the interaction between the pelvic anlagen and axial tissues medial to the pelvis, required for pelvic development [22]. The hindlimb bud was isolated at the stage prior to the appearance of the pelvic anlagen and subsequently cultured to allow skeletal development (*n* = 20/20) (figure 2). The resultant cartilaginous pelvis was perforated at the acetabulum, suggesting that the medial tissue was not necessary for acetabular perforation.

**Figure 3.**
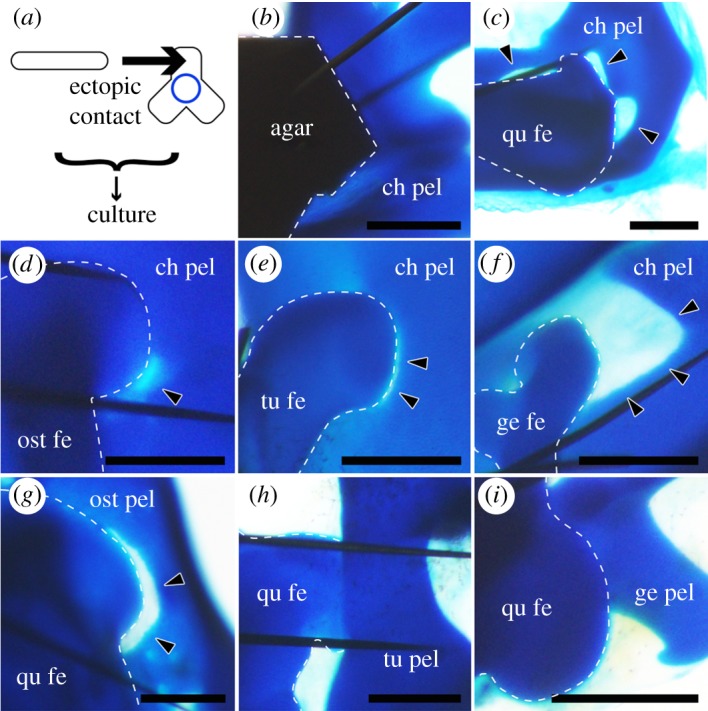
Co-culture experiments involving the femur and pelvic anlagen of several species. (*a*) Overview of the experiment. The femoral anlage and the hip interzone (rod) were ectopically articulated with the pelvic anlage (triradiate object) in a different region than the acetabulum (blue circle), and the complexed explant was cultured. (*b*) Agarose gel (agar) on the chicken pelvic anlagen (ch pel). (*c*) The quail femoral anlagen (qu fe) + the hip interzone on the chicken pelvic anlagen. (*d*) The ostrich femoral anlagen (ost fe) + the hip interzone on the chicken pelvic anlagen. (*e*) The turtle femoral anlagen (tu fe) + the hip interzone on the chicken pelvic anlagen. (*f*) The gecko femoral anlagen (ge fe) + the hip interzone on the chicken pelvic anlagen. (*g*) The quail femoral anlagen + the hip interzone on the ostrich pelvic anlagen (ost pel). (*h*) The quail femoral anlagen + the hip interzone on the turtle pelvic anlagen (tu pel). (*i*) The quail femoral anlagen + the hip interzone on the gecko pelvic anlagen (ge pel). Scale bars, 500 µm.

We next analysed the interaction between the pelvic anlagen and the lateral tissues: the femoral anlagen and the interzone, a mesenchymal tissue present in the joint region between the articulating skeletal elements only during the morphogenetic stage, which secretes several signalling molecules [23,24]. We isolated the femoral anlagen, to the proximal end of which the hip interzone is attached (electronic supplementary material, figure S1a,b), and placed it in contact with the isolated pelvic anlagen, in an area where no perforation occurs during normal development (figure 3*a*). When the area touched an agarose gel cube as a negative control, no apparent changes in the cartilage were observed (*n* = 0/6 for 4–5 days at 38.5°C, and *n* = 0/4 for 8 days at 34°C) (figure 3*b*). When the proximal end of the quail femur touched the chicken pelvis, cartilage loss was induced at the contact point (*n* = 2/3) (figure 3*c*, arrowheads), suggesting that contact with the femoral anlagen and/or the hip interzone is sufficient for pelvic anlagen perforation.

### 3.3. High susceptibility of the avian pelvic anlagen to contact with skeletal elements is responsible for acetabular perforation

To identify the tissue (pelvic anlagen, or femoral anlagen and hip interzone) responsible for the pelvic cartilage loss, we performed similar contact experiments involving the anlagen of the more distantly related species and skeletal elements other than hip joint components.

When the chicken pelvis touched the proximal end of ostrich, turtle or gecko femora, cartilage loss was induced at the contact point in every combination (*n* = 3/3, 4/8 and 8/14, respectively) (figure 3*d–f*). On the other hand, when the proximal end of the quail femora touched the ostrich, turtle or gecko pelves, only the former led to cartilage loss at the contact point (*n* = 2/4), while the turtle and gecko pelves did not (*n* = 0/24 and 0/41, respectively) (figure 3*g–i*). Since it is difficult to clarify stage correspondence among distantly related species, we used pelves from a wide range of stages of ‘reptiles’ (from the stage before pelvic development (TK11, NR6) to the stage of mature cartilage (TK19, NR24)) of both turtle and gecko, to correspond to those of birds. These observations suggest that while the femoral anlagen and hip interzones of all sauropsids have the ability to induce cartilage loss on avian pelves, the susceptibility of the pelvic anlagen is higher in birds than in ‘reptiles’.

**Figure 4.**
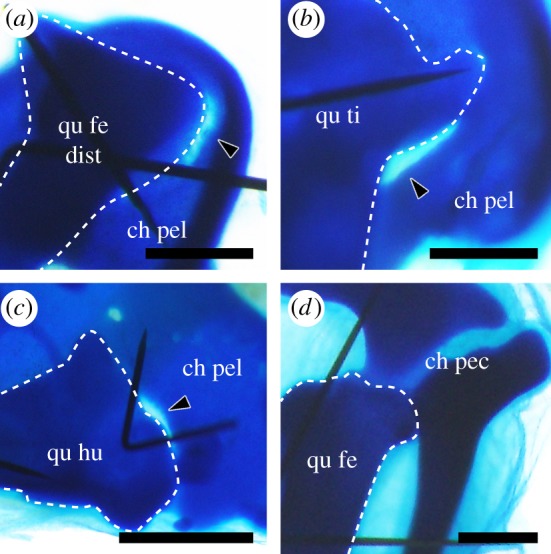
Co-culture experiments involving several skeletal elements. (*a*) The distal end of the quail femoral anlagen (qu fe dist) + the knee interzone on the chicken pelvic anlagen (ch pel). (*b*) The quail tibia anlagen (qu ti) + the knee interzone on the chicken pelvic anlagen. (*c*) The quail humerus anlagen (qu hu) + the shoulder interzone on the chicken pelvic anlagen. (*d*) The quail femur anlagen (qu fe) + the hip interzone on the chicken pectoral girdle anlagen (ch pec). Scale bars, 0.5 mm.

Next, we performed similar experiments with joint components other than the hip, namely, knee joint components (the distal end of the femur and the proximal end of the tibia) and shoulder joint components (the proximal end of the humerus and pectoral girdle). Unlike the hip joint, neither the knee nor the shoulder joint is perforated during normal development. When the chicken pelvis touched the distal end of the quail femur, the proximal end of the quail tibia or the proximal end of the quail humerus, cartilage loss was induced at the contact point (*n* = 2/5, 4/7 and 3/5, respectively) (figure 4*a–c*). On the other hand, when the chicken pectoral girdle touched the proximal end of the quail femur, cartilage loss was not induced at the contact point (*n* = 0/4) (figure 4*d*). These observations suggest that the ability to induce cartilage loss in avian pelves is not specific to hip joint components (the femoral anlagen or the hip interzone), and that the high susceptibility to it in birds is specific to the pelvis among girdles. This indicates that the avian pelvis is highly susceptible to some factor or factors, which leads to acetabular perforation.

### 3.4. Avian pelvic anlagen are more susceptible than ‘reptilian’ pelvic anlagen to hip joint-secreted molecules

**Figure 5.**
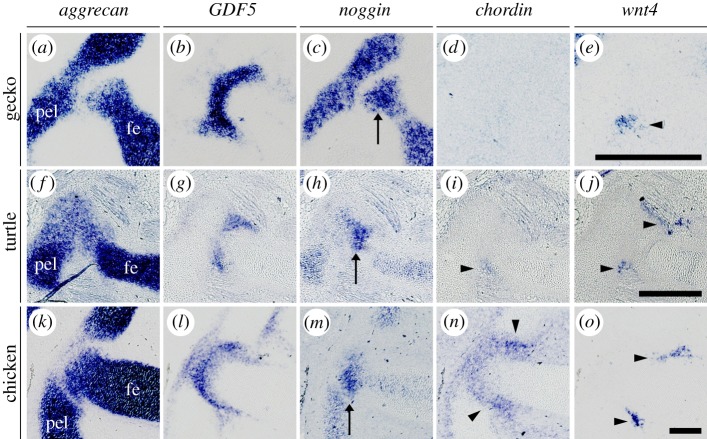
Gene expression analysis of the anti-chondrogenic paracrine factors in the embryonic hip joint. Expression of *aggrecan* (*a,f,k*; cartilage marker), *GDF5* (*b,g,l*; interzone marker), *noggin* (*c,h,m*), *chordin* (*d,i,n*) and *wnt4* (*e,j,o*) in NR18 gecko (*a–e*), TK16 turtle (*f–j*) and HH30 chicken (*k–o*). Gecko chordin signal was detected in the notochord in the same sections (not shown). pel: pelvis; fe: femur; arrows: the femoral head signal; arrowheads: the hip interzone signal. Scale bars, 300 µm.

To delineate the actual mechanism of acetabular perforation, we further focused on secretory molecules. We analysed the gene expression patterns of candidate molecules expressed in the joint region, focusing mainly on bone morphogenetic protein (BMP) antagonists (noggin and chordin) [25,26] and a Wnt ligand (wnt4) [27,28]. Antagonization of the BMP signalling pathway [29,30] and activation of Wnt signalling pathway [28,31–33] are known to suppress chondrogenesis by altering the direction of cell differentiation, and the latter pathway is crucial for joint development. Gene expression in the hip joint region was evaluated in our ‘reptile’ and bird taxa. We found the expression of noggin in the gecko femoral anlagen (Aggrecan+) (figure 5*a,c*) and wnt4 in a part of its hip interzone (GDF5+) (figure 5*b,e*), but no expression of chordin in the hip region (figure 5*d*). In the turtle hip region, we found the expression of noggin in the femoral anlagen (figure 5*f,h*), chordin and wnt4 in a part of its interzone (figure 5*g,i,j*). In the chicken hip region, we found the expression of noggin in the femoral anlagen (figure 5*k,m*), chordin in the entire interzone (figure 5*l,n*) and wnt4 in a part of its interzone (figure 5*l,o*).

**Figure 6.**
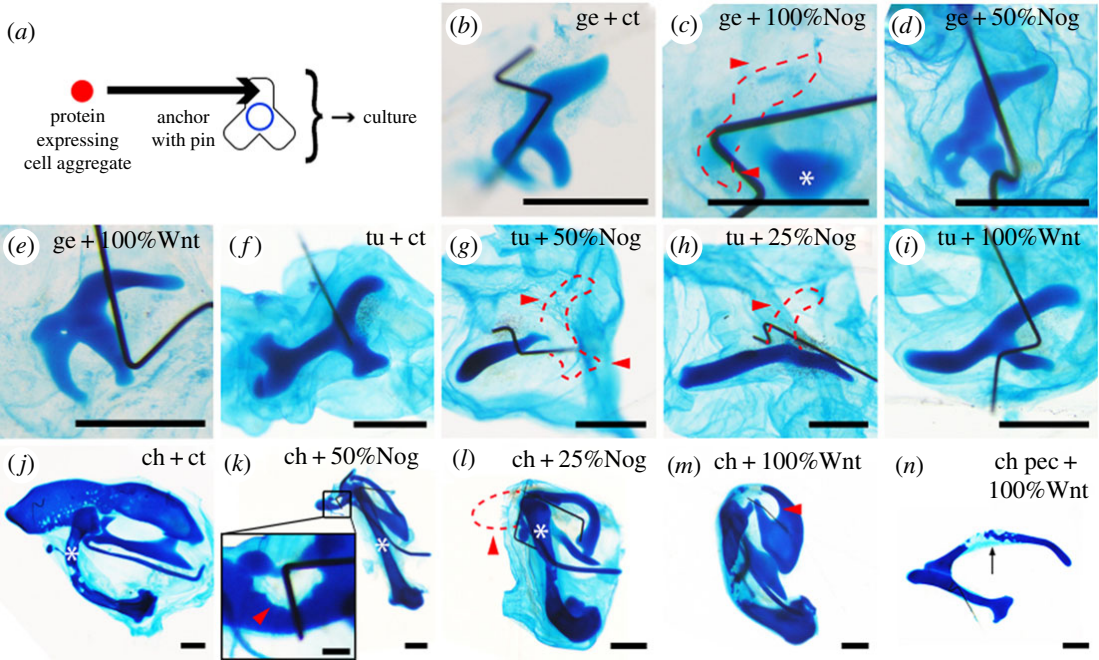
Co-culture experiments involving specific molecule-expressing cell aggregates and pelvic or pectoral anlagen of several species. (*a*) Overview of the experiment. The molecule-expressing cell aggregate (red circle) was anchored with a pin on a girdle anlage (triradiate object) in a different region than the acetabulum (blue circle), and the complexed explant was cultured. Cell aggregates (*b*) without paracrine molecule vector (control), secreting (*c*) Noggin (100%), (*d*) Noggin (50%) and (*e*) Wnt9a (100%) on the gecko pelvic anlagen. Cell aggregates (*f*) without paracrine molecule vector (control), secreting (*g*) Noggin (50%), (*h*) Noggin (25%) and (*i*) Wnt9a (100%) on the turtle pelvic anlagen. Cell aggregates (*j*) without paracrine molecule vector (control), secreting (*k*) Noggin (50%), (*l*) Noggin (25%) and (*m*) Wnt9a (100%) on the chicken pelvic anlagen. Cell aggregates secreting (*n*) Wnt9a (100%) on the chicken pectoral girdle anlagen. The percentage indicates the proportion of Noggin- or Wnt9a-expressing cells in cell aggregates, while the rest consists of cells without the paracrine molecule vector. Red dashed lines and arrowheads: cartilage loss at the cell pellet-anchoring point; asterisk: the femoral anlagen; arrow: ossification centre. Scale bars, 1 mm, except for that in the inset of *k* (200 µm).

In addition, we compared the susceptibility of the gecko, turtle and chicken pelvic anlagen to the secreted molecules. We generated cell lines expressing Gap43-TdTomato (fluorescent protein) alone (control); Gap43-TdTomato and Noggin; or Gap43-TdTomato and Wnt9a. Cell aggregates containing different ratios of these cells were prepared, and they were co-cultured with the pelvic anlagen (figure 6*a*). Cartilage loss was rarely observed when Gap43–TdTomato-only-expressing cell aggregates interacted with the gecko, turtle and chicken pelvic anlagen (*n* = 0/3, 0/3 and 1/6, respectively) (figure 6*b,f,j*). The gecko pelvic anlagen almost lost its cartilage upon incubation with the 100% Noggin cell aggregate (*n* = 3/5) but not with the 50% Noggin cell aggregate (*n* = 0/4) (figure 6*c,d*) (percentages indicate the proportion of Noggin-expressing cells in an aggregate). Conversely, the turtle and chicken pelvic anlagen lost their cartilage upon exposure to aggregates containing a lower ratio of Noggin-expressing cells: 50% (*n* = 2/3) and 25% (*n* = 4/5) for turtle (figure 6*g,h*), and 50% (*n* = 4/4) and 25% (*n* = 4/4) for chicken (figure 6*k,l*). This suggests that the turtle and chicken pelvic anlagen are more susceptible to BMP antagonization than the gecko pelvic anlagen.

Regarding the Wnt signal, 100% Wnt9a cell aggregates did not cause cartilage loss in the pelvic anlagen in gecko (*n* = 0/3) and turtle (*n* = 0/8), in contrast to chicken (*n* = 5/8) (figure 6*e,i,m*). Since it is difficult to clarify stage correspondence among distantly related species, we used pelves from a wide range of developmental stages of turtle (from the stage before pelvic development (TK15) to the stage of mature cartilage (TK18)) to correspond to those of chicken. These results suggest that the avian pelvic anlagen are more susceptible to the Wnt ligand than those of ‘reptiles’, and that differences in Wnt ligand susceptibility underpin acetabular perforation. Since 100% Wnt9a cell aggregates did not cause cartilage loss in the chicken pectoral girdle anlagen (*n* = 0/5) (figure 6*n*), Wnt ligand susceptibility might be specific to the pelvic anlagen in chicken.

### 3.5. The secretory tissue of the hip joint responsible for acetabular perforation is the interzone

**Figure 7.**
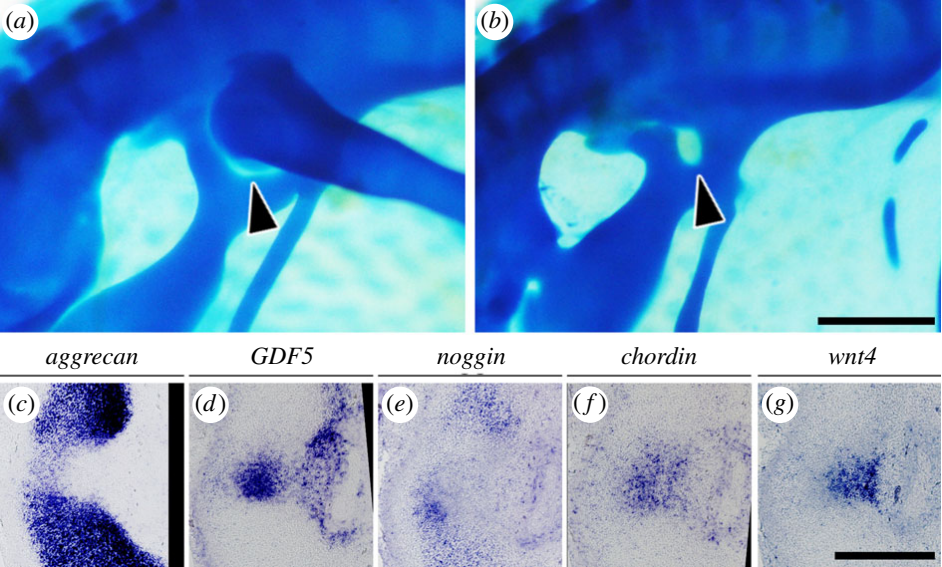
Phenotypes of limbless chicken mutants. Whole-mount alcian blue staining of (*a*) heterozygous and (*b*) homozygous HH36 embryos. (*c–g*) Gene expression analysis of cartilage, the interzone marker and anti-chondrogenic paracrine factors in the hip joint of an e7 homozygote. Lateral is to the right, and dorsal is to the top. Arrowheads: acetabular foramina. Scale bars: (*a,b*), 1 mm; (*c–g*), 300 µm.

To determine whether the femoral anlagen or the hip interzone was responsible for acetabular perforation, we analysed a limbless chicken mutant that develops the pelvis but not a distal appendicular skeleton, including femur [34]. The mutant possesses a perforated acetabulum and the hip interzone but lacks the femoral anlagen (figure 7*a–d*), which suggests that the femoral anlagen is unnecessary and the interzone is the secretory tissue involved in acetabular perforation.

Furthermore, the hip interzone of the limbless chicken mutant expressed chordin and wnt4 but not noggin (figure 7*e–g*). These findings are consistent with the hypothesis that the hip interzone and wnt4 secreted from it are responsible for acetabular perforation.

## 4. Discussion

The perforated acetabulum has long attracted scientific attention as one of the traits suggesting a close relationship between dinosaurs and extant birds [35] and as one of the most iconic synapomorphies of dinosaurs [36–40]. However, although considerable efforts have been devoted to the analysis of this trait in terms of functional morphology [1,3], almost no hypotheses have been proposed regarding its morphogenetic mechanism. A notable exception is a study by Charig [1], who speculated that the dinosaurian perforated acetabulum is linked to ossification failure. Charig postulated that the dinosaurian acetabular region consisted of cartilage that did not fossilize, resulting in an osteological gap. However, this is incompatible with the findings of the current study indicating that the dinosaur-type perforated acetabulum arises from acetabular cartilage loss in chicken embryos, which is consistent with several studies on extant birds [41,42].

**Figure 8.**
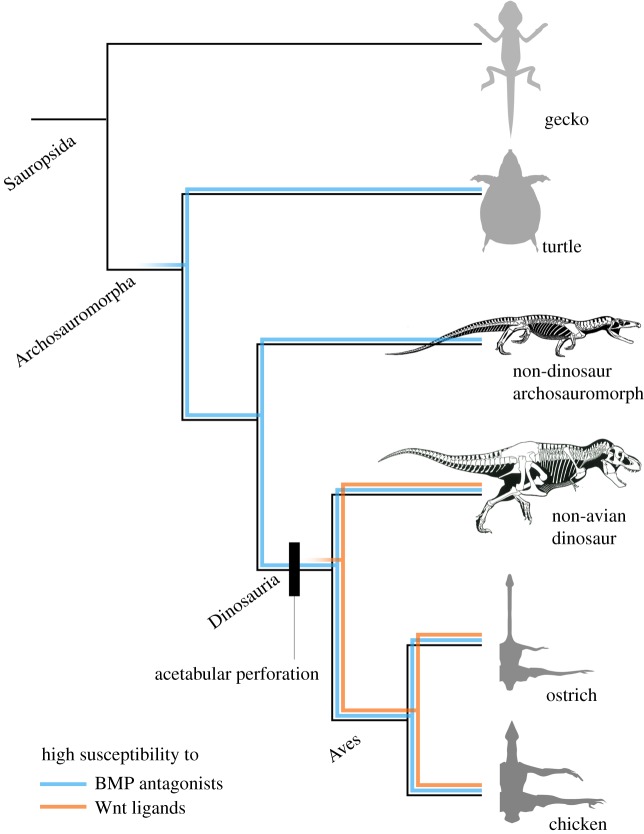
Evolutionary developmental scenario for the acquisition of acetabular perforation along the dinosaurian phylogeny. The pelvic anlagen became susceptible to BMP antagonists (blue line) during the transition from non-archosauromorph sauropsid to non-dinosaur archosauromorph, and to Wnt ligands (orange line) to induce cartilage loss at the emergence of dinosaurs, resulting in acetabular perforation. Skeletons are reprinted from [2,8] with permission.

We propose the following scenario for the acquisition of acetabular perforation in the dinosaurian lineage (figure 8). During the transition from non-archosauromorph sauropsids to dinosaurs, all embryonic hip joints secreted BMP antagonists and Wnt ligands, but the susceptibility of the pelvic anlagen to these molecules altered as follows: (1) In non-archosauromorph sauropsids (unperforated adult acetabulum), the pelvic anlagen was not susceptible to hip joint-secreted molecules, leading to no cartilage loss. (2) During the transition to non-dinosaurian archosauromorphs (unperforated adult acetabulum), the pelvic anlagen became susceptible to BMP antagonists but remained less susceptible to Wnt ligands, leading to no cartilage loss. Although one basal dinosauromorph, *Marasuchus lilloensis* [43], reportedly possessed partially perforated acetabula, it was pointed out that this partially perforated state was probably due to damage during fossilization and excavation [12,37]. Hence, this state (low susceptibility of the pelvic anlagen leading to an unperforated acetabulum) could be relevant to all non-dinosaurian archosauromorphs. (3) At the emergence of dinosaurs (perforated adult acetabulum), the pelvic anlagen became sufficiently susceptible to Wnt ligands to induce cartilage loss, resulting in acetabular perforation. This high susceptibility to the Wnt ligand was only acquired in the pelvic girdles, but not the pectoral girdles.

The detailed mechanisms underlying acetabular perforation, such as the relation between BMP antagonization and Wnt activation, remain to be elucidated. Nevertheless, the potential role of the high susceptibility of the pelvic anlagen to BMP antagonists in acetabular perforation is intriguing because it might have been acquired by basal archosauromorphs (figure 8) and might be the basis for multiple convergent acquisitions of the perforated acetabulum in Archosauria [40,44–46]. This is not unreasonable because BMP activation promotes chondrogenesis and inhibits joint development [30,47–49].

The elucidation of the mechanisms for the acquisition of Wnt ligand susceptibility in the avian pelvic anlagen is the next challenge. It might be underlain by differences in inter-tissue interaction such as heterochrony and heterotopy or by highly conserved genomic element(s), specific to birds discovered by a previous study [50]. Molecular and experimental embryological analyses will shed light on this issue.

Another challenging issue concerns the detailed analysis of fossil records to test the aforementioned hypothetical scenario. At a glance, in most basal dinosaurs, the acetabula are perforated but the femoral head shape is mismatched [1,51–53], which cannot be explained only by the mechanism we elucidate here (interaction of the pelvic anlagen with secretory molecules from the interzone) and would require auxiliary hypotheses, e.g. partial ossification failure similar to Charig's hypothesis [1], and a sophisticated methodology for scientific inference of the morphogenesis in extinct organisms.

## 5. Conclusion

In the current study, we suggest that a transiently existing cell population (the hip interzone) and secretory molecules (BMP antagonists and Wnt ligands)—both of which appear only during embryogenesis—are involved in the morphological evolution of perforated acetabula in dinosaurs. The pelvic anlagen might have cryptically evolved its susceptibility during the transition from basal sauropsids to basal archosauromorphs, although both of their acetabula are morphologically in a symplesiomorphic state (unperforated). These would have been impossible to report without analyses based on molecular and experimental embryology. Therefore, following some remarkable previous studies [54–57], we demonstrated that our knowledge of dinosaur evolution, especially the mechanistic aspect of the morphological evolution of a functionally crucial trait (dinosaur-type hip joint) can be enriched and deepened by new insights gained through an evo-devo study, particularly in experimental embryology.

## Supplementary Material

Figure S1. The hip joint inerzone.

## Supplementary Material

Table S1. Primers used for cloning

## Data accessibility

The datasets supporting this article have been uploaded as part of the electronic supplementary material.
