# Supplementary material for: Morphogenetic mechanism of the acquisition of the dinosaur-type acetabulum
Source: R Soc Open Sci. 2018 Oct 17;5(10):180604. doi: 10.1098/rsos.180604 (PMC6227947; doi:10.1098/rsos.180604)
Supplement: Figure S1. The hip joint inerzone. [file rsos180604supp1.docx]

**Supplementary information**

**
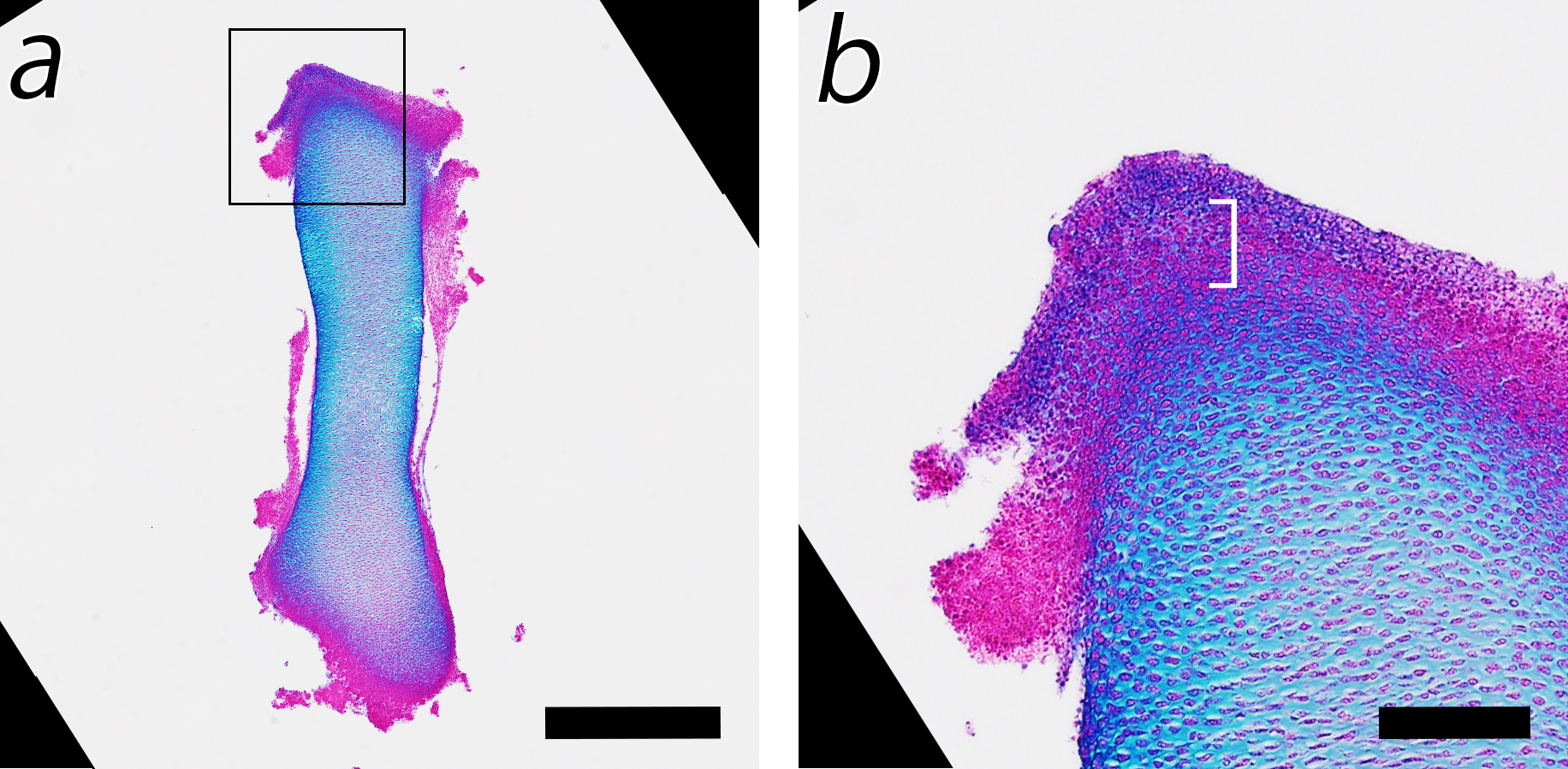
**

Figure S1. The hip joint interzone. (a) Histological section of an isolated HH30 (the stage we used for co-culture experiments) chicken femoral anlagen. (b) The magnification of the interzone region. Square, the region magnified in b; square bracket in b, the hip joint interzone. Scale bars, 0.5 mm in a, 0.1 mm in b.
