# Supplementary material for: Morphogenetic mechanism of the acquisition of the dinosaur-type acetabulum
Source: R Soc Open Sci. 2018 Oct 17;5(10):180604. doi: 10.1098/rsos.180604 (PMC6227947; doi:10.1098/rsos.180604)
Supplement: Table S1. Primers used for cloning [file rsos180604supp2.docx]

**Table S1. Primers used for cloning**

|  | 5′ | 3′ |
| --- | --- | --- |
| chicken-noggin | ATCATTCCCAGTGCCTTGTG | TAAATGCACGGACTTGGCAG |
| chicken-chordin | ATGCGCACCGCCCTGCTGCTGCTCGC | TCAGCGGCTCCATGCCTCTGCTGTGGCT |
| chicken-wnt4 | ATGAGCCCGGAGTATTTCCTGCGCTCCTTG | ACCGGCACGTGTGGATTTCCACCACCCGATG |
| turtle-GDF5 | AGCGGGAACTCTACTGGACT | GTGGGATTGTGCTGCTACCT |
| turtle-noggin | TGACTCTGTACGCCTTGGTG | GGTCAAATGCACCGACTTGG |
| turtle-chordin | GCTCCTGGTCCAAGTCCTTC | CAACGATCGGGCGTATCTCA |
| turtle-wnt4 | AAAGGGTTAATCCAGCGCCA | GTCCTCATCCGTGTGTGGTT |
| gecko-noggin | ATGGATCATTCCCCGGGGGCTGTGACTC | CTAGCACGAGCATTTGCACTCGCC |
| gecko-wnt4 | CTTTGGAAAGGTGGTCACGC | GGTCTTGTTGCATTGCCTCC |
